# Synthesis and Biological Evaluation of 2-Thioxopyrimidin-4(1*H*)-one Derivatives as Potential Non-Nucleoside HIV-1 Reverse Transcriptase Inhibitors

**DOI:** 10.3390/ijms151120723

**Published:** 2014-11-12

**Authors:** Nagy M. Khalifa, Mohamed A. Al-Omar

**Affiliations:** 1 Pharmaceutical Chemistry Department, Drug Exploration & Development Chair, College of Pharmacy, King Saud University, Riyadh 11451, Saudia Arabia; E-Mail: malomar1@ksu.edu.sa; 2 Therapeutical Chemistry Department, Pharmaceutical and Drug Industries Division, National Research Centre, Dokki 12622, Cairo, Egypt

**Keywords:** AIDS, non-nucleoside reverse transcriptase inhibitors (NNRTIs), 2-alkylthio-6-benzylpyrimidin-4(3H)-ones (S-DABOs), anti-HIV-1 activity

## Abstract

A series of new 5-allyl-6-benzylpyrimidin-4(3*H*)-ones bearing different substituents at the C-2 position of the pyrimidine core have been synthesized and evaluated for their *in vitro* activities against human immunodeficiency virus type 1 (HIV-1) in the human T-lymphotropic type (MT-4 cell cultures). The majority of the title compounds showed moderate to good activities against HIV-1. Amongst them, 5-allyl-6-benzyl-2-(3-hydroxypropylthio)pyrimidin-4(3*H*)-one analogue **11c** exhibited the most potent anti-HIV-1 activity (IC_50_ 0.32 µM). The biological testing results clearly indicated that the substitution at C-2 position of the pyrimidine ring could increase the anti-HIV-1 reverse transcriptase (RT) activity.

## 1. Introduction

The reverse transcriptase (RT) of the human immunodeficiency virus type 1 (HIV-1 RT) is one of the main targets of drugs used in the treatment of AIDS [1,2]. Several RT inhibitors have been developed and approved by the FDA and are currently in clinical use. Among several kinds of anti-HIV agents, non-nucleoside reverse transcriptase inhibitors (NNRTIs), specifically targeting to reverse transcriptase, have attracted wide attention due to their high specificity, excellent potency and low cytotoxicity [3,4]. NNRTIs are a class of antiretroviral drugs which deactivate HIV-1 reverse transcriptase by inducing conformational change of the enzyme via binding to a hydrophobic site close (approximately 10 Å) to its catalytic site [5]. Since these compounds do not need intracellular metabolic activation, have relatively low cytotoxicity, act on a nanomolar scale and are not active against other retroviruses, they are highly specific HIV-1 inhibitors. Among NNRTIs, dihydro alkoxy benzyl oxopyrimdines (DABOs) are an interesting class of compounds active at nanomolar concentration. This class was first reported in 1992 and further developed during the following years into S-DABOs, N-DABOs and related analogues [6,7,8,9,10,11,12,13,14,15,16,17,18,19,20,21,22,23,24,25,26]. Over the past decade, 2-alkylthio-6-benzylpyrimidin-4(3*H*)-ones (S-DABOs) have been the subject of great interest and have led to the identification of several new structures [27,28,29] displaying excellent activities as non-nucleoside inhibitors of HIV-1 reverse transcriptase (RT). In our continuing efforts to find novel effective and selective anti-HIV-1 agents in 2-alkylsulfanyl-6-benzyl-3,4-dihydropyrimidin-4(3*H*)-ones (S-DABOs) type NNRTIs [30,31,32,33], herein we present the synthesis and anti-HIV-1 activities of a series of S-DABO analogs.

## 2. Results and Discussion

### 2.1. Chemistry

**Scheme 1:**
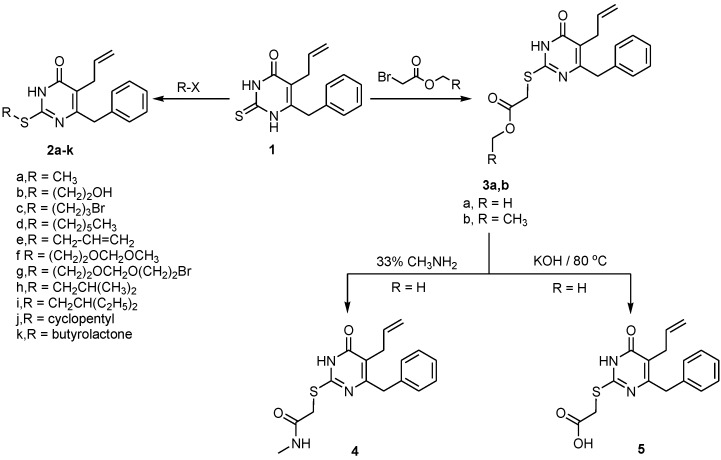
Synthetic route of *S*-alkyl derivatives **2**–**5**.

**Scheme 2:**
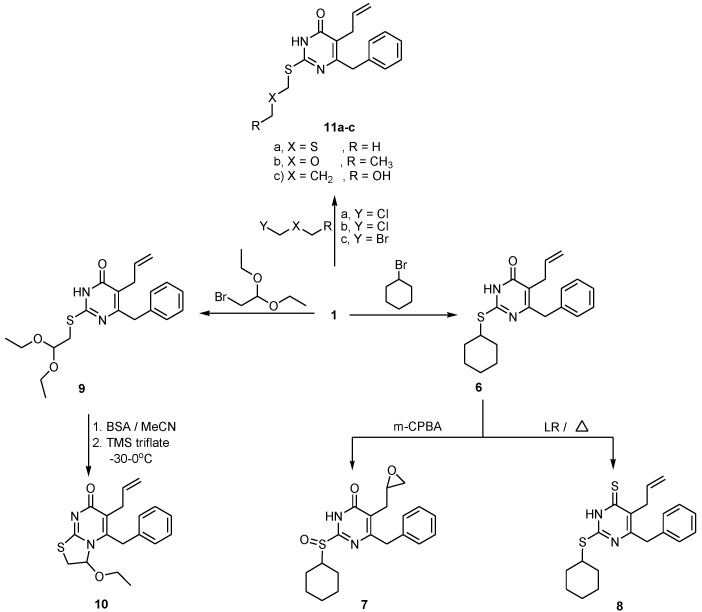
Synthetic route of *S*-substituted and fused pyrimidin-4(3*H*)-one derivatives **6**–**11**.

The synthetic route of the newly designed compounds is described in Scheme 1 and Scheme 2. The key intermediate 5-allyl-6-benzyl-2,3-dihydro-2-thioxopyrimidin-4(1*H*)-one (**1**) was prepared through the method of our previously reported 3-step synthetic strategy [23] through the reaction of β-ketoester with thiourea in the presence of EtONa in refluxing EtOH. Subsequent S-alkylation of **1** with appropriate alkyl halides namely, methyl iodide, 1-bromoethanol, 1,3-dibrompropane, 1-bromohexane, allyl iodide, 1-bromo-2-(methoxymethoxy)ethane, bis(2-bromoethoxy)methane, methyl bromoacetate, ethyl bromoacetate, isobutyl bromide, 1-bromo-2-ethyl butane, cyclopentyl bromide, α-bromo-γ-butyrolactone, chloroethyl methyl ether and 3-bromo-1-prapanol in dry dimethylformamide (DMF) in the presence of anhydrous K_2_CO_3_ afforded the desirable target S-DABO analogues **2a-k**. 5-Allyl-6-benzyl-2-(methyl acetatethio) pyrimidin-4(3*H*)-one **3a** and 5-Allyl-6-benzyl-2-(ethyl acetatethio)pyrimidin-4(3*H*)-one **3b** were obtained when compound **1** was treated with various halo-esters namely, methyl bromoacetate or ethyl bromoacetae in anhydrous DMF in the presence of K_2_CO_3_, respectively. The amide **4** was obtained upon treatment of **3a** in 33% ethanolic methylamine. Also, the ester **3a** was hydrolyzed to the corresponding acid **5** in hot aqueous potassium hydroxide (Scheme 1).

However, treatment of compound **1** with cyclohexyl bromide, afforded *S*-cycloalkyl derivative **6**, which upon oxidation with *m*-chloroperbenzoic acid gave the corresponding cyclohexylsulfinyl derivative **7**. Treatment of compound **6** with 2,4-bis(4-methoxyphenyl)-1,3-dithia-2,4-diphosphetan-2,4-disulfide (Lawesson’s Reagent) in anhydrous benzene afforded 5-allyl-6-benzyl-2-(cyclohexylthio)pyrimidine-4(3*H*)-thione **8** (Scheme 2).

Furthermore, reaction of **1** with 2-bromoacetaldehyde acetal in anhydrous DMF and K_2_CO_3_ at 65 °C gave the corresponding 5-allyl 6-benzyl-2-(2,2-diethoxyethylthio)pyrimidin-4(3*H*)-one **9** which was then treated with *N*,*O*-bis(trimethylsilyl) acetamide (BSA) using trimethylsilyl trifluoromethane-sulfonate (TMS triflate) as a catalyst to yield 5-(3,5-dimethylbenzyl)-6-allyl-3-ethoxy-2,3-dihydrothiazolo-[3,2-a]pyrimidin-7-one **10**. Acyclic nucleoside Compounds **11a-c** were obtained by *S*-alkylation of the key intermediate 2-thioxopyrimidin-4(1*H*)-one **1** with different acyclic halosugars (Scheme 2). All the target compounds were characterized by nuclear magnetic resonance (NMR), mass spectrum (MS) and elemental analysis. Both analytical and spectral data of all the newly prepared compounds are in full agreement with the proposed structures.

### 2.2. Pharmacological Screening

The anti-HIV activities and cytotoxicity’s of the synthesized S-DABOs analogues are summarized in Table 1. The expression of HIV-1 was quantified by two different methods, either the HIV-1 antigen detection assay enzyme-linked immunosorbent assay (ELISA) [34] or indirectly by the 3-(4,5-dimethylthiazolyl-2)-2,5-diphenyltetrazolium bromide (MTT) assay [35]. In general, the activity of the tested compounds including S-DABOs derivatives in the side chains at C-2 is to be expected when compared to those previously reported for S-DABOs. As illustrated in Table 1, we could see that most of those compounds showed moderate to potent inhibition of RT activity, suggesting that HIV-1 RT was the target of this series of S-DABO derivatives. The activity and cytotoxicity of these newly synthesized S-DABO analogues were evaluated in MT-4 cells for their ability to inhibit HIV-1 in MT-4 cells,in comparison with (6-benzyl-1-(ethoxymethyl)-5-isopropylpyrimidine-2,4-dione (MKC-442) and azidothymidine (AZT) used as reference drugs. Most of the tested compounds exhibited moderate to good activities against wild-type HIV-1 with EC_50_ values ranged from 35.00 to 0.32 µM. The most active compound **11c** showed the highest activity against wild-type HIV-1 with an EC_50_ value of 0.32 µM. The other compounds displayed moderate activity **2e**, **2j**, EC_50_ 2.5 and 2.60 µM, respectively or were inactive **2c**, **2k**, **3b**, **4** and **9**, against HIV-1 replication.

**Table 1:** Cytotoxicity and anti-HIV-1 activity of newly synthesized compounds in MT-4 cells.

Compound	IC_50 _^a ^(µM)	CC_50 _^b ^(µM)	SI ^c^
**1**	–	–	–
**2a**	26.8	>100	>3
**2c**	–	–	–
**2d**	7.0	35.0	>5
**2e**	2.5	25.0	>10
**2h**	1.0	>100	>100
**2j**	2.6	>100	>38
**2k**	–	–	–
**3a**	35.0	>100	>2
**3b**	–	–	–
**4**	–	–	–
**8**	3.9	>100	>25
**9**	–	–	–
**11a**	3.16	>100	>31
**11c**	0.32	>100	>312
**MKC-442**	0.005	141	28,000
**AZT**	0.04	52	1,300

^a^ IC_50_, 50% inhibitory concentration; ^b^ CC_50_, 50% cytotoxic concentration; ^c^ Selectivity index (SI), CC_50_/IC_50_.

## 3. Experimental Section

### 3.1. Chemistry

All melting points were uncorrected and determined on a Büchi melting point apparatus (Hamburg, Germany). NMR spectra were recorded on a Varian Gemini 2000 NMR spectrometer (Varian Inc., Palo Alto, CA, USA) at 300 MHz for ^1^H NMR and 75 MHz for ^13^C NMR using tetramethylsilane (TMS) as internal standard. All chemical shifts are quoted in δ values using parts per million scale (ppm) downfield from TMS at the University of Southern Denmark, Denmark. EI mass spectra were recorded on a Finnigan MAT SSQ 710 spectrometer (Finnigan MAT, Madison, WI, USA) at 70 eV at the University of Southern Denmark, Denmark. Elemental analyses were performed at Atlantic Micro lab, Inc., Atlanta, GA, USA, and the found values agreed favourably with the calculated ones. The progress of reactions was monitored by TLC (analytical silica gel plates 60 F_254_). Merck silica gel (0.040–0.063 mm) was used for column chromatography. The analytical spectral analyses were supported by Erik B. Pedersen, University of Southern Denmark, Institute of Chemistry, and antiviral screening *in vitro* through the Retrovirus Laboratory, Department of Virology, State Serum Institute, Copenhagen, Denmark.

***5-Allyl 6-(benzyl)-2-(methylthio)pyrimidin-4(3H)-one* (2a)**. A mixture of compound **1** (1.29 mmol), 10 mL hexamethyldisilazane (HMDS) and 10 mg (NH_4_)_2_SO_4_ were refluxed for 2 h. The remaining HMDS was removed under reduced pressure. 1 mL of anhydrous DMF and (2.24 mmol) of methyl iodide were added and the solution was stirred at room temperature for 5 h. 100 mL of dichloromethane was added and the solution was washed with 20 mL anhydrous NaHCO_3_. The organic layer was dried over Na_2_SO_4_ and evaporated till dryness under reduced pressure. The solid was washed with benzene to remove iodide and the solid was dissolved in CH_2_Cl_2_, dried and purified by column chromatography (30% EtOAc/pet.ether 60–80 °C) to give compound **2a** in 94% yield, m.p. 214–216 °C; ^1^H NMR (DMSO-d_6_) δ: 2.38 (s, 3H, CH_3_), 3.18 (d, 2H, CH_2_), 3.80 (s, 2H, CH_2_Ph), 4.94 (m, 2H, =CH_2_), 5.75 (m, 1H, CH), 7.15–7.26 (m, 5H, ArH), 12.58 (s, 1H, NH exchangeable with D_2_O); ^13^C NMR (DMSO-d_6_) δ: 12.50, 28.49, 39.40, 115.29, 116.05, 126.30, 128.57, 129.10, 134.12, 138.26, 158.39, 160.97, 163.17; MS *m*/*z* (%): 272 (M^+^). Analytical for C_15_H_16_N_2_OS (272.37): calculated C 66.15, H 5.92, N 10.29; found C 66.07, H 5.83, N 10.23.

***5-Allyl 6-(benzyl)-2-(alkylthio)pyrimidin-4(3H)-one* (2b-k, 11b,c)**. General procedure: To a stirred solution of compound **1** (3 mmol) in anhydrous DMF (10 mL) was added K_2_CO_3_ (3.3 mmol) and appropriate alkyl or cycloalkyl halides (3.3 mmol). The stirring was continued at room temperature for 8–12 h. The reaction mixture was poured into cold H_2_O (150 mL), the resulting precipitate was collected by filtration and washed sequentially with small portions of H_2_O, MeOH, Et_2_O and dried *in vacuo* to afford the corresponding crude product, which was purified by flash chromatography to give the pure target compounds.

***2-(2-Hydroxyethylthio)-5-allyl-6-benzylpyrimidin-4(3H)-one* (2b)**. Yield 76%, m.p. 164–166 °C (30% EtOAc/pet.ether 60–80 °C); ^1^H NMR (DMSO-d_6_) δ: 3.01 (s, 2H, CH_2_), 3.12 (d, 2H, CH_2_), 3.81 (s, 2H, CH_2_Ph), 4.28 (t, 2H, CH_2_), 4.96 (m, 2H, =CH_2_), 5.73 (m, 1H, CH), 7.05–7.19 (m, 5H, ArH), 12.36 (s, 1H, NH exchangeable with D_2_O); ^13^C NMR (DMSO-d_6_) δ: 28.51, 32.68, 39.42, 63.22, 115.26, 116.08, 126.34, 128.55, 129.11, 134.16, 138.24, 158.41, 161.03, 163.21; MS *m*/*z* (%): 302 (M^+^). Analytical for C_16_H_18_N_2_O_2_S (302.39): calculated C 63.55, H 6.00, N 9.26; found C 63.41, H 5.87, N 9.11.

***2-(3-Bromopropylthio)-5-allyl-6-benzylpyrimidin-4(3H)-one* (2c)**. Yield 66%, m.p. 138–140 °C (30% EtOAc/pet.ether 60–80 °C); ^1^H NMR (DMSO-d_6_) δ: 2.47 (m, 2H, CH_2_), 2.96 (t, 2H, SCH_2_), 3.11 (d, 2H, CH_2_), 3.45 (t, 2H, SCH_2_), 3.82 (s, 2H, CH_2_Ph), 4.95 (m, 2H, =CH_2_), 5.78 (m, 1H, CH), 7.18–7.25 (m, 5H, ArH); ^13^C NMR (DMSO-d_6_) δ: 24.89, 28.15, 31.09, 32.48, 39.40, 115.34, 117.89, 126.36, 128.49, 129.12, 134.28, 137.50, 154.71, 160.83, 163.67; MS *m*/*z* (%): 298 (M^+^). Analytical for C_17_H_19_BrN_2_OS (379.31): calculated C 53.83, H 5.05, N 7.39; found C 53.76, H 4.86, N 7.23.

***5-Allyl-6-benzyl-2-(hexylthio)pyrimidin-4(3H)-one* (2d)**. Yield 73%, m.p. 153–155 °C (30% EtOAc/pet.ether 60–80 °C); ^1^H NMR (DMSO-d_6_) δ: 0.98 (t, 3H, CH_3_), 1.26–1.95 (4m, 8H, 4CH_2_), 2.98 (t, 2H, SCH_2_), 3.17 (d, 2H, CH_2_), 3.86 (s, 2H, CH_2_Ph), 5.01 (m, 2H, =CH_2_), 5.73 (m, 1H, CH), 7.16–7.24 (m, 5H, ArH), 12.48 (s, 1H, NH exchangeable with D_2_O); ^13^C NMR (DMSO-d_6_) δ: 14.25, 23.46, 26.43, 27.65, 28.41, 30.89, 32.07, 39.42, 115.29, 117.85, 126.37, 128.52, 129.16, 134.22, 137.58, 155.26, 161.12, 163.69; MS *m*/*z* (%): 342 (M^+^). Analytical for C_20_H_26_N_2_OS (342.50): calculated C 70.14, H 7.65, N 8.18; found C 70.11, H 7.49, N 8.05.

***5-Allyl 6-benzyl-2-(allylthio)pyrimidin-4(3H)-one* (2e)**. Yield 69%, m.p. 114–116 °C (30% EtOAc/pet.ether 60–80 °C); ^1^H NMR (DMSO-d_6_) δ: 3.16 (d, 2H, SCH_2_), 3.21 (d, 2H, CH_2_), 3.89 (s, 2H, CH_2_Ph), 4.91 (m, 2H, =CH_2_), 5.04 (m, 2H, =CH_2_), 5.76 (m, 1H, CH), 5.05 (m, 1H, CH), 7.21–7.26 (m, 5H, ArH), 12.35 (s, 2H, NH exchangeable with D_2_O); ^13^C NMR (DMSO-d_6_) δ: 28.68, 32.43, 34.15, 39.41, 115.73, 117.90, 126.59, 128.64, 129.41, 134.85, 135.23, 137.82, 154.75, 161.49, 163.90; MS *m*/*z* (%): 298 (M^+^). Analytical for C_17_H_18_N_2_OS (298.40): calculated C 68.42, H 6.08, N 9.39; found C 68.29, H 6.01, N 9.28.

***5-Allyl-6-benzyl-2-(2-(methoxymethoxy)ethylthio)pyrimidin-4(3H)-one* (2f)**. Yield 69%, m.p. 188–190 °C (30% EtOAc/pet.ether 60–80 °C); ^1^H NMR (DMSO-d_6_) δ: 3.06 (t, 2H, CH_2_), 3.21 (d, 2H, CH_2_), 3.76 (s, 3H, CH_3_), 3.85 (s, 2H, CH_2_Ph), 4.27 (t, 2H, CH_2_), 5.01 (m, 2H, =CH_2_), 5.56 (s, 2H, CH_2_), 5.70 (m, 1H, CH), 7.15–7.21 (m, 5H, ArH), 12.47 (s, 1H, NH exchangeable with D_2_O); ^13^C NMR (DMSO-d_6_) δ: 28.33, 31.22, 39.51, 56.01, 67.99, 98.65, 115.32, 117.49, 126.55, 128.61, 129.34, 134.97, 137.62, 154.42, 161.19, 163.52; MS *m*/*z* (%): 346 (M^+^). Analytical for C_18_H_22_N_2_O_3_S (346.44): calculated C 62.40, H 6.40, N 8.09; found C 62.34, H 6.29, N 7.95.

***5-Allyl-6-benzyl-2-(2-((2-bromoethoxy)methoxy)ethylthio)pyrimidin-4(3H)-one* (2g)**. Yield 59%, m.p. 192–194 °C (3% MeOH/CHCl_3_); ^1^H NMR (DMSO-d_6_) δ: 3.10 (t, 2H, CH_2_), 3.16 (d, 2H, CH_2_), 3.51 (t, 2H, CH_2_), 3.84 (s, 2H, CH_2_Ph), 4.09, 4.17 (2t, 4H, 2CH_2_), 5.05 (m, 2H, =CH_2_), 5.60 (s, 2H, CH_2_), 5.78 (m, 1H, CH), 7.19–7.24 (m, 5H, ArH), 12.47 (s, 1H, NH exchangeable with D_2_O); ^13^C NMR (DMSO-d_6_) δ: 27.39, 28.35, 30.12, 39.56, 67.92, 69.01, 95.86, 115.30, 117.53, 126.50, 128.67, 129.32, 134.91, 137.59, 154.38, 160.87, 163.41; MS *m*/*z* (%): 439 (M^+^). Analytical for C_19_H_23_BrN_2_O_3_S (439.37): calculated C 51.94, H 5.28, N 6.38; found C 51.82, H 5.19, N 6.32.

***5-Allyl-6-benzyl-2-(isobutylthio)pyrimidin-4(3H)-one* (2h)**. Yield 69%, m.p. 134–136 °C (30% EtOAc/pet.ether 60–80 °C); ^1^H NMR (DMSO-d_6_) δ: 0.98 (d, 6H, 2CH_3_), 2.18 (m, 1H, CH), 2.98 (d, 2H, CH_2_), 3.17 (d, 2H, CH_2_), 3.72 (s, 2H, CH_2_Ph), 5.01 (m, 2H, =CH_2_), 5.87 (m, 1H, CH), 7.16–7.23 (m, 5H, ArH), 12.58 (s, 1H, NH exchangeable with D_2_O); ^13^C NMR (DMSO-d_6_) δ: 21.69, 30.21, 28.10, 39.44, 40.75, 115.48, 117.91, 126.34, 128.52, 129.17, 134.31, 137.52, 154.69, 161.09, 163.59; MS *m*/*z* (%): 314 (M^+^). Analytical for C_18_H_22_N_2_OS (314.45): calculated C 68.75, H 7.05, N 8.91; found C 68.70, H 6.94, N 8.85.

***5-Allyl-6-benzyl-2-(2-ethylbutylthio)pyrimidin-4(3H)-one* (2i)**. Yield 72%, m.p. 141–143 °C (30% EtOAc/pet.ether 60–80 °C); ^1^H NMR (DMSO-d_6_) δ: 0.96 (t, 6H, 2CH_3_), 1.32 (m, 4H, 2CH_2_), 2.05 (m, 1H, CH), 2.95 (d, 2H, CH_2_), 3.11 (d, 2H, CH_2_), 3.83 (s, 2H, CH_2_Ph), 4.99 (m, 2H, =CH_2_), 5.82 (m, 1H, CH), 7.19–7.26 (m, 5H, ArH), 12.61 (s, 1H, NH exchangeable with D_2_O); ^13^C NMR (DMSO-d_6_) δ: 11.35, 27.01, 28.19, 34.92, 39.46, 41.76, 115.41, 117.87, 126.30, 128.55, 129.14, 134.28, 137.49, 155.03, 160.78, 163.65; MS *m*/*z* (%): 342 (M^+^). Analytical for C_20_H_26_N_2_OS (342.5): calculated C 70.14, H 7.65, N 8.18; found C 70.12, H 7.59, N 8.10.

***5-Allyl 6-benzyl-2-(cyclopentylthio)pyrimidin-4(3H)-one* (2j)**. Yield 65%, m.p. 117–119°C (30% EtOAc/pet.ether 60–80 °C); ^1^H NMR (DMSO-d_6_) δ: 1.53–2.29 (m, 8H, 4CH_2_), 3.21 (d, 2H, CH_2_), 3.70 (m, 1H, CH), 3.91 (s, 2H, CH_2_Ph), 4.95 (m, 2H, =CH_2_), 5.74 (m, 1H, CH), 7.12–7.20 (m, 5H, ArH), 12.28 (s, 1H, NH exchangeable with D_2_O); ^13^C NMR (DMSO-d_6_) δ: 26.19, 28.19, 32.21, 34.64, 39.50, 115.62, 117.89, 126.33, 128.52, 129.17, 134.24, 137.64, 154.55, 161.21, 163.75; MS *m*/*z* (%): 326 (M^+^). Analytical for C_19_H_22_N_2_OS (326.46): calculated C 69.90, H 6.79, N 8.58; found C 69.84, H 6.70, N 8.49.

***5-Allyl-6-benzyl-2-(tetrahydro-2-oxofuran-3-ylthio)pyrimidin-4(3H)-one* (2k)**. Yield 72%, m.p. 153–155 °C (30% EtOAc/pet.ether 60–80 °C); ^1^H NMR (DMSO-d_6_) δ: 2.63 (m, 2H, CH_2_), 3.21 (d, 2H, CH_2_), 3.83 (s, 2H, CH_2_Ph), 4.12 (t, 1H, CH), 4.34 (t, 2H, CH_2_), 4.96 (m, 2H, =CH_2_), 5.82 (m, 1H, CH), 7.16–7.28 (m, 5H, ArH), 12.54 (s, 1H, NH exchangeable with D_2_O); ^13^C NMR (DMSO-d_6_) δ: 28.73, 29.48, 39.85, 41.32, 66.29, 115.64, 118.24, 126.38, 128.47, 129.10, 134.51, 137.80, 154.42, 163.18, 165.00, 173.73; MS *m*/*z* (%): 342 (M^+^). Analytical for C_18_H_18_N_2_O_3_S (342.41): calculated C 63.14, H 5.30, N 8.18; found C 63.05, H 5.22, N 8.11.

***5-Allyl-6-benzyl-2-(methylacetatethio)pyrimidin-4(3H)-one* (3a)**. Yield 78%, m.p. 117–119 °C (30% EtOAc/pet.ether 60–80 °C); ^1^H NMR (DMSO-d_6_) δ: 3.12 (d, 2H, CH_2_), 3.76 (s, 3H, CH_3_), 3.80 (s, 2H, CH_2_Ph), 3.95 (s, 2H, CH_2_), 5.02 (m, 2H, =CH_2_), 5.87 (m, 1H, CH), 7.12–7.20 (m, 5H, ArH), 12.51 (s, 1H, NH exchangeable with D_2_O); ^13^C NMR (DMSO-d_6_) δ: 28.16, 32.10, 39.48, 56.14, 115.62, 117.91, 126.38, 128.54, 129.19, 134.31, 137.43, 154.72, 161.17, 163.69, 170.23; MS *m*/*z* (%): 330 (M^+^). Analytical for C_17_H_18_N_2_O_3_S (330.4): calculated C 61.80, H 5.49, N 8.48; found C 61.68, H 5.41, N 8.36.

***5-Allyl-6-benzyl-2-(ethylacetatethio)pyrimidin-4(3H)-one* (3b)**. Yield 61%, m.p. 118–120 °C (30% EtOAc/pet.ether 60–80 °C); ^1^H NMR (DMSO-d_6_) δ: 1.28 (t, 3H, CH_3_), 3.09 (d, 2H, CH_2_), 3.84 (s, 2H, CH_2_), 3.89 (s, 2H, CH_2_Ph), 4.27 (q, 2H, CH_2_), 5.05 (m, 2H, =CH_2_), 5.83 (m, 1H, CH), 7.15–7.22 (m, 5H, ArH), 12.38 (s, 1H, NH exchangeable with D_2_O); ^13^C NMR (DMSO-d_6_) δ: 14.35, 28.19, 31.70, 39.42, 61.48, 115.66, 117.87, 126.32, 128.56, 129.13, 134.26, 137.49, 154.63, 161.22, 163.73, 169.80; MS *m*/*z* (%): 344 (M^+^). Analytical for C_18_H_20_N_2_O_3_S (344.43): calculated C 62.77, H 5.85, N 8.13; found C 62.65, H 5.74, N 7.96.

***2-(5-Allyl-4-benzyl-1,6-dihydro-6-oxopyrimidin-2-ylthio)-N-methylacetamide* (4)**. The ester **3a** (0.60 mmol) was dissolved in 33% methylamine in EtOH (10 mL). When TLC showed no more starting material, the solution was evaporated *in vacuo* to dryness to give the amide derivative **4** in 100% yield, m.p. 167–168 °C; ^1^H NMR (DMSO-d_6_) δ: 2.51 (s, 2H, CH_3_), 3.16 (d, 2H, CH_2_), 3.74 (s, 2H, CH_2_), 3.78 (s, 2H, CH_2_Ph), 4.93 (m, 2H, =CH_2_), 5.73 (m, 1H, CH), 7.16–7.25 (m, 5H, ArH), 7.88, 12.46 (2s, 2H, 2NH exchangeable with D_2_O); ^13^C NMR (DMSO-d_6_) δ: 25.85, 28.62, 33.39, 39.51, 115.32, 116.87, 126.35, 128.51, 129.16, 135.34, 138.27, 158.34, 161.21, 163.82, 171.85; MS *m*/*z* (%): 329 (M^+^). Analytical for C_17_H_19_N_3_O_2_S (344.43): calculated C 61.98, H 5.81, N 12.76; found C 61.87, H 5.76, N 12.71.

***2-(5-Allyl-4-benzyl-1,6-dihydro-6-oxopyrimidin-2-ylthio)acetic acid* (5)**. The ester **3a** (0.9 mmol) was dissolved in hot (80 °C) aqueous potassium hydroxide (3 mL), and the solution was kept at this temperature for 5 min. After cooling, water was added (3 mL) and the pH adjusted to 7 with 4 M HCl. The precipitate was collected, washed with cold water and dried to give the acid derivative **5** in 61% yield, m.p. 172–173 °C; ^1^H NMR (DMSO-d_6_) δ: 3.23 (d, 2H, CH_2_), 3.79 (s, 2H, CH_2_), 3.85 (s, 2H, CH_2_Ph), 4.97 (m, 2H, =CH_2_), 5.83 (m, 1H, CH), 7.17–7.28 (m, 5H, ArH), 12.60, 12.73 (2s, 2H, OH and NH exchangeable with D_2_O); ^13^C NMR (DMSO-d_6_) δ: 28.09, 31.95, 39.75, 115.98, 117.11, 126.39, 128.47, 129.15, 134.10, 137.20, 156.15, 160.73, 163.75, 169.55; MS *m*/*z* (%): 316 (M^+^). Analytical for C_17_H_19_N_3_O_2_S (316.37): calculated C 60.74, H 5.10, N 8.85; found C 60.67, H 5.01, N 8.76.

***5-Allyl 6-benzyl-2-(cyclohexylthio)pyrimidin-4(3H)-one* (6)**. To a stirred solution of compound **1** (3 mmol) in anhydrous DMF (10 mL) was added K_2_CO_3_ (3.3 mmol) and cyclohexyl bromide (3.3 mmol). The stirring was continued at 80 °C for 6 h. The reaction mixture was poured into cold H_2_O (100 mL), the resulting precipitate was collected by filtration and dried *in vacuo* to afford the corresponding crude product, which was purified by flash chromatography (30% EtOAc/pet.ether 60–80 °C) to give compound **6** in 56% yield, m.p. 138–140 °C; ^1^H NMR (DMSO-d_6_) δ: 1.20–1.84 (m, 10H, 5CH_2_), 3.23 (d, 2H, CH_2_), 3.79 (m, 1H, CH), 3.87 (s, 2H, CH_2_Ph), 4.98 (m, 2H, =CH_2_), 5.76 (m, 1H, CH), 5.85 (m, 1H, CH), 7.14–7.29 (m, 5H, ArH), 12.49 (s, 1H, NH exchangeable with D_2_O); ^13^C NMR (DMSO-d_6_) δ: 24.97, 25.55, 28.51, 32.26, 39.43, 43.13, 115.31, 116.84, 126.32, 128.50, 129.14, 135.39, 138.45, 157.13, 161.48, 162.93; MS *m*/*z* (%): 340 (M^+^). Analytical for C_20_H_24_N_2_OS (340.48): calculated C 70.55, H 7.10, N 8.23; found C 70.47, H 7.03, N 8.12.

***6-Benzyl-2-(cyclohexylsulfinyl)-5-((oxiran-2-yl)methyl)pyrimidin-4(3H)-one* (7)**. A solution of compound **6** (2.5 mmol) and m-chloroperbenzoic acid (3 mmol) in dry dichloromethane (20 mL) was allowed to stand at room temperature for 48 h. The reaction mixture was diluted with chloroform (100 mL) and successively washed with 10% sodium bisulfite, 10% sodium thiosulfate, saturated sodium bicarbonate and water. After evaporation, the product was purified by preparative TLC (silica gel, CH_2_Cl_2_/MeOH 30:1) to give the title compound **7** in 37% yield, m.p. 190–192 °C; ^1^H NMR (DMSO-d_6_) δ: 1.43–2.12 (m, 10H, 5CH_2_), 2.27 (d, 2H, CH_2_), 3.07 (m, 1H, CH), 3.28 (m, 1H, CH), 3.76 (d, 2H, CH_2_), 3.85 (s, 2H, CH_2_Ph), 7.19–7.26 (m, 5H, ArH), 12.58 (s, 1H, NH exchangeable with D_2_O); ^13^C NMR (DMSO-d_6_) δ: 21. 09, 23.15, 25.44, 25.84, 34.03, 39.93, 46.64, 61.93, 115.13, 126.31, 128.43, 129.14, 137.45, 154.17, 161.59, 164.28; MS *m*/*z* (%): 372 (M^+^). Analytical for C_20_H_24_N_2_O_3_S (372.48): calculated C 64.49, H 6.49, N 7.52; found C 64.41, H 6.43, N 7.45.

***5-Allyl-6-benzyl-2-(cyclohexylthio)pyrimidine-4(3H)-thione* (8)**. A mixture of compound **6** (0.61 mmol) and 2,4-bis(4-methoxyphenyl)-1,3-dithia-2,4-diphosphetan-2,4-disulfide (Lawesson’s Reagent) (0.92 mmol) in anhydrous benzene 50 mL was refluxed for 1 h. After cooling, the solvent was removed under reduced pressure and the precipitate formed was purified by passing through a silica gel column chromatography eluting with (20% CHCl_3_/pet.ether) to afford the title compound **8** in 53% yield, m.p. 143–145 °C; ^1^H NMR (DMSO-d_6_) δ: 1.31–1.98 (m, 10H, 5CH_2_), 3.42 (d, 2H, CH_2_), 3.77 (m, 1H, CH), 3.82 (s, 2H, CH_2_Ph), 5.05 (m, 2H, =CH_2_), 5.91 (m, 1H, CH), 7.21–7.36 (m, 5H, ArH), 12.59 (s, 1H, NH exchangeable with D_2_O); ^13^C NMR (DMSO-d_6_) δ: 25.85, 26.32, 29.36, 33.07, 40.63, 44.49, 115.75, 117.89, 126.61, 128.51, 129.62, 135.34, 138.46, 157.73, 163.35, 179.47; MS *m*/*z* (%): 356 (M^+^). Analytical for C_20_H_24_N_2_S_2_ (356.55): calculated C 67.37, H 6.78, N 7.86; found C 67.28, H 6.781, N 7.79.

***5-Allyl 6-benzyl-2-(2,2-diethoxyethylthio)pyrimidin-4(3H)-one* (9)**. The same procedure as mentioned in compound **6**, except the stirring was continued at 65 °C for 8 h. Yield 65%, m.p. 156–158 °C (30% EtOAc/pet.ether 60–80 °C); ^1^H NMR (DMSO-d_6_) δ: 1.08 (t, 6H, 2CH_3_), 3.12 (d, 2H, SCH_2_), 3.22 (d, 2H, CH_2_), 3.59 (q, 4H, 2 OCH_2_), 3.94 (s, 2H, CH_2_Ph), 4.52 (t, 1H, CH), 5.03 (m, 2H, =CH_2_), 5.78 (m, 1H, CH), 7.18–7.23 (m, 5H, ArH), 12.52 (s, 1H, NH exchangeable with D_2_O); ^13^C NMR (DMSO-d_6_) δ: 15.01, 28.51, 32.47, 39.45, 61.72, 101.35, 115.34, 117.33, 126.51, 128.67, 129.38, 135.36, 137.70, 154.72, 161.28, 163.55; MS *m*/*z* (%): 374 (M^+^). Analytical for C_20_H_26_N_2_O_3_S (374.5): calculated C 64.14, H 7.00, N 7.48; found C 64.05, H 6.87, N 7.39.

***6-Allyl-5-benzyl-3-ethoxy-2,3-dihydrothiazolo[3,2-a]pyrimidin-7-one* (10)**. Compound **9** (4 mmol) was dissolved in dry MeCN (10 mL) under Argon and *N*,*O*-bis(trimethylsilyl) acetamide (4.4 mmol) was added. The mixture was cooled to −40 °C, and TMS-triflate (4 mmol) was added drop wise. The mixture was allowed to warm to room temperature slowly (typically overnight). Cold saturated aqueous NaHCO_3_ was added, and the mixture was extracted with CH_2_Cl_2_ (3 × 20 mL). The organic phase was washed with saturated aqueous NaCl, dried, evaporated *in vacuo* and purified by column chromatography [CHCl_3_/pet.ether (60–80 °C)] to give the title compound **10** in 46% yield, m.p. 162–164 °C; ^1^H NMR (DMSO-d_6_) δ: 1.12 (t, 3H, CH_3_), 3.06 (d, 2H, CH_2_), 3.31 (d, 2H, CH_2_), 3.54 (q, 2H, CH_2_), 3.80 (s, 2H, CH_2_Ph), 3.94 (t, 1H, CH), 4.88 (m, 2H, =CH_2_), 5.65 (m, 1H, CH), 7.15–7.34 (m, 5H, ArH); ^13^C NMR (DMSO-d_6_) δ: 14.38, 29.55, 30.32, 33.30, 39.43, 61.96, 89.74, 115.46, 117.91, 126.59, 128.46, 129.67, 135.68, 146.45, 166.77, 168.28; MS *m*/*z* (%): 328 (M^+^). Analytical for C_18_H_20_N_2_O_2_S (328.43): calculated C 65.83, H 6.14, N 8.53; found C 65.71, H 6.03, N 8.47.

***2-((Methylthio)methylthio)-5-allyl-6-benzylpyrimidin-4(3H)-one* (11a)**. A mixture of compound **1** (1.29 mmol), 10 mL HMDS and 10 mg (NH_4_)_2_SO_4_ were refluxed for 2 h. After evaporation under vacuum, the silylated compound was dissolved in 20 mL anhydrous MeCN and stirred at −40 °C. (4.0 mmol) of chloromethylmethyl sulfide was added, followed by dropwise addition of 0.40 mL TMS-triflate in 10 mL of anhydrous MeCN. The temperature was raised gradually to −15 °C and the mixture was kept at this temperature overnight, then the temperature was raised 5 °C per half hour. The reaction was stopped at 5 °C and 100 mL of CH_2_Cl_2_ was added. The mixture was washed with 20 mL saturated NaHCO_3_ and the organic layer was dried over Na_2_SO_4_ and evaporated till dryness under reduced pressure. The crude product was purified by flash chromatography (3% MeOH/CHCl_3_) to give the title product **11a** in 74% yield, m.p. 218–220 °C; ^1^H NMR (DMSO-d_6_) δ: 2.11 (s, 3H, CH_3_), 3.33 (d, 2H, CH_2_), 3.91 (s, 2H, CH_2_Ph), 4.25 (s, 2H, CH_2_), 5.10 (m, 2H, =CH_2_), 5.86 (m, 1H, CH), 7.20–7.31 (m, 5H, ArH), 12.53 (s, 1H, NH exchangeable with D_2_O); ^13^C NMR (DMSO-d_6_) δ: 15.29, 28.97, 36.38, 40.27, 115.73, 118.39, 126.53, 128.61, 129.17, 134.69, 137.87, 156.50, 163.08, 165.22; MS *m*/*z* (%): 318 (M^+^). Analytical for C_16_H_18_N_2_OS_2_ (318.46): calculated C 60.34, H 5.70, N 8.80; found C 60.28, H 5.62, N 8.69.

***5-Allyl-6-benzyl-2-(ethoxymethylthio)pyrimidin-4(3H)-one* (11b)**. Yield 73%, m.p. 142–144 °C (2% MeOH/CHCl_3_); ^1^H NMR (DMSO-d_6_) δ: 1.10 (t, 3H, CH_3_), 3.21 (d, 2H, SCH_2_), 3.45 (q, 2H, CH_2_), 3.90 (s, 2H, CH_2_Ph), 5.05 (m, 2H, =CH_2_), 5.33 (s, 2H, CH_2_), 5.80 (m, 1H, CH) 7.23–7.29 (m, 5H, ArH), 12.31 (s, 2H, NH exchangeable with D_2_O); ^13^C NMR (DMSO-d_6_) δ: 14.50, 29.02, 40.24, 64.92, 72.25, 115.77, 118.93, 126.55, 128.58, 129.32, 135.11, 137.91, 155.75, 161.77, 164.65; MS *m*/*z* (%): 316 (M^+^). Analytical for C_16_H_18_N_2_OS_2_ (316.42): calculated C 64.53, H 6.37, N 8.85; found C 64.45, H 6.29, N 8.76.

***2-(3-Hydroxypropylthio)-5-allyl-6-benzylpyrimidin-4(3H)-one* (11c)**. Yield 66%, m.p. 123–125 °C (3% MeOH/CHCl_3_); ^1^HNMR (DMSO-d_6_) δ: 2.17 (m, 2H, CH_2_), 2.94 (t, 2H, SCH_2_), 3.14 (d, 2H, CH_2_), 3.56 (t, 2H, SCH_2_), 3.84 (s, 2H, CH_2_Ph), 4.58 (s broad, 1H, OH), 4.98 (m, 2H, =CH_2_), 5.87 (m, 1H, CH), 7.12–7.21 (m, 5H, ArH), 12.38 (s, 1H, NH exchangeable with D_2_O); ^13^CNMR (DMSO-d_6_) δ: 22.65, 28.37, 30.12, 39.46, 62.03, 115.36, 117.93, 126.42, 128.51, 129.15, 134.22, 137.55, 154.70, 161.02, 163.65; MS *m*/*z* (%): 316 (M^+^). Analytical for C_17_H_20_N_2_O_2_S (316.42): calculated C 64.53, H 6.37, N 8.85; found C 64.37, H 6.325, N 8.73.

### 3.2. Pharmacological Screening

#### 3.2.1. Virus and cells

The HIV-1 strains HTLV-IIIB and NNRTI resistant strain N 119 were propagated in H9 cells at 37 °C, 5% CO_2_ using RPMI 1640 with 10% heat-inactivated fetal calf serum (FCS) and antibiotics (growth medium). The culture supernatant was filtered (0.45 nm), liquated and stored at −80 °C until use. Both HIV-1 strains were obtained from the NIH AIDS Research and Reference program.

#### 3.2.2. Inhibition of Human Immunodeficiency Virus Type 1 (HIV-1) Replication

Compounds were examined for possible antiviral activity against both strains of HIV-1 using MT-4 cells as target cells. MT4 cells were incubated with virus (0.005 MOI) and growth medium containing the test dilutions of compounds for six days in parallel with virus-infected and uninfected control cultures without compound added. Expression of HIV in the cultures was quantitated by the HIV-1 antigen detection assay ELISA [27] or indirectly quantified using the MTT assay [28]. Compounds mediating less than 30% reduction of HIV expression were considered without biological activity. Compounds were tested in parallel for cytotoxic effect in uninfected MT-4 cultures containing the test dilutions of compound as described above. A 30% inhibition of cell growth relative to control cultures was considered significant. The 50% inhibitory concentrations (IC_50_) and the 50% cytotoxic concentrations (CC_50_) were determined by interpolation from the plots of percent inhibition *vs.* concentration of compound.

## 4. Conclusions

Non-nucleoside reverse transcriptase inhibitors (NNRTI) are key components in highly active antiretroviral therapy for treating HIV-1. Herein we present the synthesis for a series of S-DABOs analogues and evaluated as non-nucleoside HIV-1 reverse transcriptase inhibitors for inhibition of HIV-1 replication. Various chemical and spectral data supported the structures of the newly synthesized compounds. The majority of the tested compounds showed moderate to good activities against HIV-1 with an IC_50_ range from 35.60 to 0.32 µM. These results provided useful indicators for guiding the further rational design of new S-DABO analogues as more active and selective HIV-1 inhibitors.
